# Electronic and magnetic properties of SnO_2_/CrO_2 _thin superlattices

**DOI:** 10.1186/1556-276X-6-146

**Published:** 2011-02-15

**Authors:** Pablo D Borges, Luísa MR Scolfaro, Horácio W Leite Alves, Eronides F da Silva, Lucy VC Assali

**Affiliations:** 1 Instituto de Física, Universidade de São Paulo, CP 66318, São Paulo, SP, 05315-970, Brazil; 2 Department of Physics, Texas State University, San Marcos, TX, 78666, USA; 3 Universidade Federal de São João Del Rei, CP 110, São Joao Del Rei, MG, 36301-160, Brazil; 4 Departamento de Fisica, Universidade Federal de Pernambuco, Recife, PE, 50670-901, Brazil

## Abstract

In this article, using first-principles electronic structure calculations within the spin density functional theory, alternated magnetic and non-magnetic layers of rutile-CrO_2 _and rutile-SnO_2 _respectively, in a (CrO_2_)_*n*_(SnO_2_)_*n *_superlattice (SL) configuration, with *n *being the number of monolayers which are considered equal to 1, 2, ..., 10 are studied. A half-metallic behavior is observed for the (CrO_2_)_*n*_(SnO_2_)_*n *_SLs for all values of *n*. The ground state is found to be FM with a magnetic moment of 2 μ_B _per chromium atom, and this result does not depend on the number of monolayers *n*. As the FM rutile-CrO_2 _is unstable at ambient temperature, and known to be stabilized when on top of SnO_2_, the authors suggest that (CrO_2_)_*n*_(SnO_2_)_*n *_SLs may be applied to spintronic technologies since they provide efficient spin-polarized carriers.

## Introduction

A variety of heterostructures have been studied for spintronics applications, and they have proved to have a great potential for high-performance spin-based electronics [1]. A key requirement in developing most devices based on spins is that the host material must be ferromagnetic (FM) above 300 K. In addition, it is necessary to have efficient spin-polarized carriers. One approach to achieve the spin injection is to create built-up superlattices (SLs) of alternating magnetic and non-magnetic materials. One attempt has already been made by Zaoui et al. [2], through *ab initio *electronic structure calculations for the one monolayer (ZnO)_1_(CuO)_1 _SL, with the aim of obtaining a half-metallic behavior material, since they are 100% spin polarized at the Fermi level and therefore appear ideal for a well-defined carrier spin injection.

In this study, the magnetic and electronic properties of (CrO_2_)_*n*_(SnO_2_)_*n *_SLs with *n *= 1, 2, ..., 10 being the number of monolayers are investigated. These systems are good candidates to obtain a half-metallic behavior material since bulk rutile-CrO_2 _has shown experimentally this behavior [3] and recently magnetic tunnel junctions based on CrO_2_/SnO_2 _epitaxial layers have been obtained [4].

### Theoretical method

**Figure 1:**
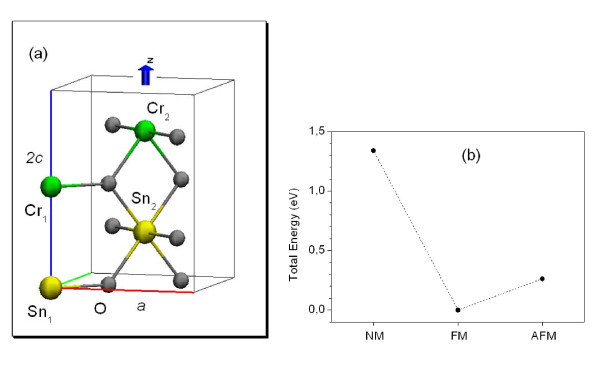
**The supercell model and total energies for the systems. (a)** Supercell used to study the (SnO_2_)_1_(CrO_2_)_1_ SL, and **(b)** Total energies for the non-magnetic (NM) and anti-ferromagnetic (AFM) states relative to the ferromagnetic (FM) state. The dashed lines connecting the points are to guide the eyes.

All the calculations were based on the spin density functional theory. The Projector-Augmented Wave method implemented in the Vienna Ab-initio Simulation Package (VASP-PAW) [5,6] was employed in this study, and for the exchange-correlation potential, the generalized gradient approximation and the Perdew, Burke, and Ernzerhof (GGA-PBE) approach was used [7]. The valence electronic distribution for the PAWs representing the atoms were Sn-- 4*d*^10 ^5*s*^2 ^5*p*^2^, Cr-- 3*d*^5 ^5*s*^1^, and O-2*s*^2 ^2p^4^. Scalar relativistic effects were included. For simulation of the one monolayer (CrO_2_)_1_(SnO_2_)_1 _SL, a supercell with 12 atoms (2Sn, 2Cr, and 8O) in the rutile structure as shown in Figure 1a was used. For this case, a 4 × 4 × 3 mesh of Monkhorst-Pack *k*-points was used for integration in the SL BZ. All the calculations were done with a 490 eV energy cutoff in the plane-wave expansions.

## Results and discussion

**Figure 2:**
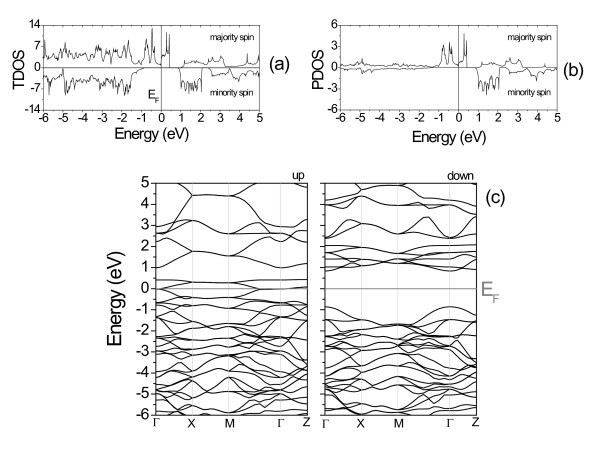
**Density of states and band structure for the (SnO_2_)_1_(CrO_2_)_1_ SL**. **(a)** Total density of states (TDOS), **(b)** Project density of states (PDOS) for the Cr-d orbital, **(c)** Band structure, for spin up and spin down, along the main symmetry lines of the SL BZ. The Fermi level, E_F_, is set to zero in (a), (b), and (c).

**Figure 3:**
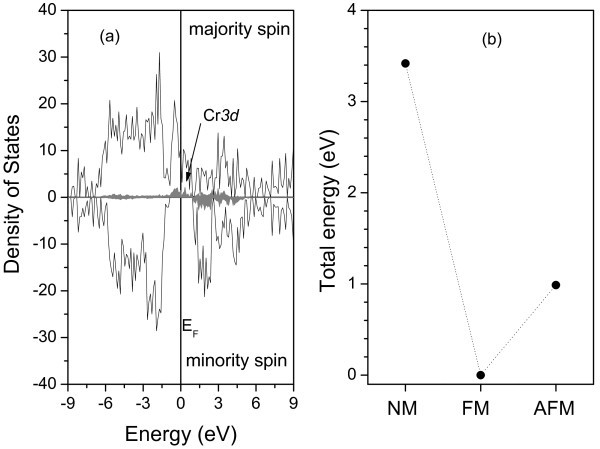
**Density of states and total energies for the SL with n=10. (a) **Total density of states (in black) and project density of states (in gray) for the Cr--3d. **(b) **Total energies for the non magnetic (NM) and anti-ferromagnetic (AFM) states relative to the ferromagnetic (FM) state. The Fermi level, E_F_, is set to zero. The dashed lines connecting the points are to guide the eyes.

For the (CrO_2_)_1_(SnO_2_)_1 _SL, the calculation was started with the experimental lattice parameters of the tin dioxide, *a *= 4.737 Å, *c/a = *0.673, and *u *= 0.307 [8-10]. The system was relaxed until the residual forces on the ions were less than 10 meV/Å. Good agreement between the calculated and the available experimental values for the lattice parameters is obtained, as seen in Table 1. Figure 1b shows that the ground state is ferromagnetic (FM), being the most stable state compared with the non-magnetic (NM) and anti-ferromagnetic (AFM) ones. For the ground state, the total magnetic moment gives a value of 2 μ_B _per chromium atom. Figure 2a,b presents the total density of states (TDOS) and the projected density of states (PDOS), respectively for the Cr 3d orbital, showing that the system has a half metallic behavior, with the Cr 3d orbital appearing in the gap region, characterizing a metallic-like behavior for the majority spin and a semiconductor-like behavior for the minority spin. The band structures of the SL for spin up and spin down are depicted in Figure 2c. A band gap of approximately 1.71 eV is obtained for the minority spin at the Г-point. There is a smaller gap for spin flip excitations from the Fermi level, which is approximately 0.86 eV. For the (SnO_2_)_*n*_(CrO_2_)_*n *_SLs with *n *>1, considered here up to *n *= 10, it was observed that the ground state remains as FM. The interplay of the SnO_2 _and CrO_2 _layer thicknesses does not change the half-metallic behavior, as can be verified through the DOS shown in Figure 3a,b for *n *= 10. The magnetic moment per Cr atom, in all the studied cases, is the same and equal to 2 μ_B_. Moreover, the SL magnetization does not depend on the number of monolayers. This has been verified by performing calculations with one monolayer of CrO_2 _grown between 3, 7, and 11 monolayers of SnO_2_. It was observed that the SL magnetization remained equal to 2 μ_B_. Our results show a 100% spin polarization at the Fermi level, ideal for a well-defined carrier spin injection.

**Table 1:** Experimental and calculated values for the lattice parameters of the SnO_2_, CrO_2_, and of the (CrO_2_)_1_(SnO_2_)_1 _and (CrO_2_)_10_(SnO_2_)_10 _SLs in the rutile structure

	*a *(Å)	*c/a*	*u*
SnO_2_	4.737^a^	0.673^a^	0.307^a^
	4.839^b^	0.670^b^	0.306^b^
CrO_2_	4.421^c^	0.6596^c^	0.301^c^
	4.455^d^	0.6569^d^	0.304^d^
(CrO_2_)_1_(SnO_2_)_1_	4.625^d^	0.658^d^	^-^
(CrO_2_)_10_(SnO_2_)_10_	4.640^d^	6.546^d^	^-^

^a^[8]; ^b^[9]; ^c^[10]; ^d^this work

**Figure 4:**
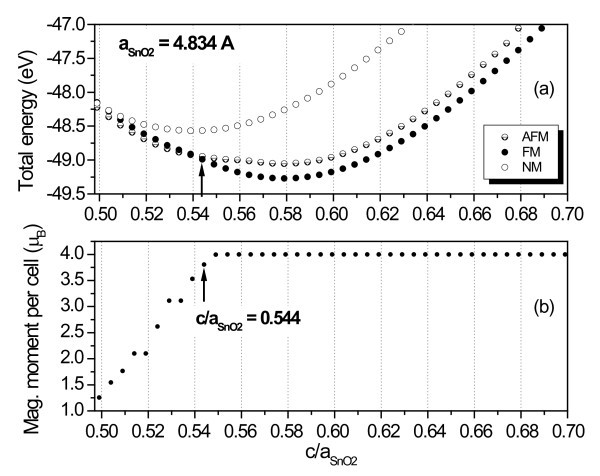
**Study of strain effects on the magnetic behavior. (a)** Total energy versus the ***c/a***_SnO2_ parameter for bulk rutile-CrO_2_ for AFM, FM, and NM states. **(b) **Magnetic moment per cell versus the c/a_SnO2_ parameter.

An investigation, related to strain effects along the *z*-direction for the rutile phase of CrO_2_, was made by simulating bulk rutile-CrO_2_, on top of tin dioxide, assuming for CrO_2 _the lattice parameter *a *of SnO_2_, i.e., a situation in which the chromium dioxide is tensile. By varying the ratio *c/a*_SnO2 _and minimizing the total energy of the system, the authors obtained the curves shown in Figure 4a for the FM, AFM, and NM states, showing that the transition from a FM to an AFM state occurs when *c/a*_SnO2 _is about 0.544. At this value, a magnetic moment reduction is observed, as depicted in Figure 4b. These results suggest a magnetization change when the SL is under strain or, in other words, when CrO_2 _is compressed. A similar behavior was found by Srivastava et al. for bulk rutile-CrO_2 _under pressure [11].

The advantage in using the SnO_2_/CrO_2 _SLs, despite the fact that CrO_2 _is unstable at room temperature, is that its stability becomes possible when grown on SnO_2 _[12]. Our results showed that the interface effects due to the lattice mismatch do not change the chromium dioxide magnetism characteristics. If the distances between two planes perpendicular to the rutile *c*-axis containing the Cr_2 _and Sn_1 _are compared (see Figure 1a), at the interface region of the SL, before and after full relaxations, then changes of only approximately 4% are observed for all the studied SLs.

## Conclusions

In conclusion, the results of first-principles electronic structure calculations, within the spin density functional theory, carried out for (CrO_2_)_*n*_(SnO_2_)_*n *_SLs formed by alternating magnetic and non-magnetic layers of rutile-CrO_2 _and rutile-SnO_2_, where the number of monolayers *n *was varied from 1 to 10, have been reported in this article. A half-metallic behavior is observed for all the studied (CrO_2_)_*n*_(SnO_2_)_*n *_SLs. The ground state is FM, with a magnetic moment of 2 μ_B _per chromium atom, which is independent of the number of monolayers. As the FM rutile-CrO_2 _is unstable at ambient temperature, and known to be stabilized when on top of SnO_2_, it is suggested that (CrO_2_)_*n*_(SnO_2_)_*n *_SLs may be applied to spintronic technologies since they provide efficient spin-polarized carriers.

## Abbreviations

AFM: anti-ferromagnetic; FM: ferromagnetic; GGA-PBE: generalized gradient approximation and the Perdew, Burke, and Ernzerhof; NM: non-magnetic; PDOS: projected density of states; SL: superlattice; TDOS: total density of states; VASP-PAW: Vienna Ab-initio Simulation Package and the Projected Augmented Wave.

## Competing interests

The authors declare that they have no competing interests.

## Authors' contributions

PB performed the ab initio calculations, participated in the analysis, and drafted the manuscript. LS and PB conceived of the study. HA, ES, LA, and LS participated in the analysis and in the production of a final version of the manuscript. All authors read and approved the final manuscript.
